# Fungal chimera: A lethal mammalian fungus with invasion strategies of plant pathogens

**DOI:** 10.1080/21505594.2024.2439497

**Published:** 2024-12-26

**Authors:** Carol Uphoff Meteyer, Justin G. Boyles

**Affiliations:** a Wildlife Pathologist United States, Sedona, AZ, USA; b School of Biological Sciences, Southern Illinois University, Carbondale, IL, USA

**Keywords:** Ascomycota, biotrophy, hemibiotrophy, pathogen emergence, pathogenic fungi, virulence factors

## Main text

A new pathogen had emerged. Hibernating bats in caves were being found dead by the thousands, but no one knew why. A conspicuous fungus on the surface of the bat’s muzzle led to the name, white-nose syndrome (WNS), but a cutaneous fungal pathogen causing mortality in mammals is rare enough that no one initially knew if this new white fungus was a cause or a symptom of WNS. The answer came soon after. The first tissues examined under a microscope provided startling evidence the fungus was in fact an ascomycete pathogen with very atypical behaviour. It invaded the skin in a way that had never before been seen by penetrating the skin surface and then changing shape as it multiplied in defined nests within the epidermis. Alarmingly, the invading fungus did not elicit visible inflammation or cell death [1] as it proliferated within the intraepidermal space. This covert invader eventually breaks out of the intraepidermal nests penetrating deep into the skin, sometimes spanning the thickness of the bat wing [1]. The fungus was identified as *Pseudogymnoascus destructans* and confirmed to be the cause of WNS [2–4]. WNS has killed millions of bats and is now considered a real threat to the survival of some species [5].

In the decade that followed these initial discoveries, there were significant advances in our understanding of the physiological and ecological effects of WNS on bats and their populations. However, a plausible and coherent understanding of the pathogenesis of *P. destructans* remained elusive. In 2022, Meteyer et al. [6] provided evidence for a unique invasion strategy not previously reported in vertebrates or invertebrates. The evidence suggested that, unlike mammalian skin fungi like athlete’s foot or ringworm, *P. destructans* does not stay on the skin surface, but invades bat skin similar to plant pathogens that devastate crops around the world (e.g. Magnaportha/Pyricularia, Sclerotinia, Botrytis).

*P. destructans* hyphae initially enter and proliferate within the intraepidermal space of bat skin without tissue damage or host cell inflammation, similar to the biotrophy used by plant fungi to penetrate and multiply within plant tissues without host detection or cell death [7]. Similar to hemibiotrophic fungal pathogens of plants that switch from biotrophy to invasive necrotrophy, *P. destructans* emerges from clandestine intraepidermal nests aggressively invading deep dermal tissue. High-resolution phylogenomic analysis provides more evidence that similarity in the invasion strategies of *P. destructans* and some plant pathogens are not coincidental. Instead, *P. destructans* is found in an ascomycete clade that is otherwise populated with plant-related fungi, including many economically significant hemibiotrophic plant fungal pathogens [6]. The findings presented by Meteyer et al. [6] questioned the lines of pathogen evolution that are drawn between the plant and animal kingdoms, but additional work was still needed to validate this unconventional hypothesis.

Against this backdrop, we read with great interest a recent study in the journal Science that also looked at the mechanisms exploited by *P. destructans* to invade bat skin [8]. Isidoro-Ayza and Klein provided additional support for the hypotheses presented in Meteyer et al. [6] and proposed some interesting pathways for future work. They first replicated microscopic and electron microscopic evidence for a two-phase invasion strategy similar to those in Meteyer et al. [6]. In Fig. 1A-C Isidoro-Ayza and Klein also show that *P. destructans* hyphae multiplies within the epidermis without inflammation or necrosis, with subsequent penetration of hyphae into the deeper tissue. Isidoro-Ayza and Klein developed a novel little brown bat (*M. lucifugus*) keratinocyte cell line (MYLUC) as a model to further study the early invasion (biotrophic phase) of *P. destructans*. Images of opaque and translucent MYLUK cells [8 Fig. 2B] also support the hypothesis by Meteyer et al. [6] that *P. destructans* uses the plant invasion strategy of biotrophy to invade without host cell death. An interesting characteristic of the hyphae depicted in Fig. 2B by Isidoro-Ayza and Klein [8] is their resemblance to appressoria, an additional invasion strategy used by plant fungal pathogens and suspected in *P. destructans* [6]. The cell line developed by Isidoro-Ayza and Klein is useful for studying the early stages of invasion by *P. destructans*, but model systems used to study the invasion strategies of plant pathogens [7] closely related to *P. destructans* may provide further insights into the invasive character of *P. destructans*, including the formation of appressoria.

Emerging diseases caused by novel pathogens or known pathogens in new hosts can have devastating effects on plants and animals [9]. In addition to providing additional evidence supporting Meteyer et al. [6], the findings in Isidoro-Ayza and Klein [8] further reinforce our suggestion [6] that much can be gained by bringing together researchers from plant and animal fields to study the emergence of pathogens. And, as a “fungal chimera” with characteristics recognized independently in dermatophytes of mammals, hemibiotrophic pathogens of plants, environmental saprophytes, and with thermal restrictions as an obligate cryophile, *P. destructans* is uniquely positioned to address seminal questions regarding pathogen evolution. Plants were on Earth before animals, and ancestors of ascomycete plant pathogens were saprophytes. Ancient genes coding for enzymes that make nutrients available (cellulase, lipase, protease) and produce cell components such as melanin are essential for the survival of both pathogens and saprophytes [10]. Conserved genes in the same species of fungus can express genes selectively, based on the characteristics of different hosts [11]. The ability of fungi to conserve the ancient strategy of cell penetration through appressoria-like structures has been proposed to exist within the genomes of fungi that do not produce these structures [12]. This is supported by their presence in fungal pathogens of plants [7] and insects [13], as well as in saprophytes [14]. The findings presented in 2022 by Meteyer et al. [6] and two years later by Isidoro-Ayza and Klein [8] document plant virulence factors in *P. destructans*. Although this lethal fungal pathogen of bats has only been studied since 2007, decades of research in the field of fungal pathogenesis in plants present a trove of information relevant to our understanding of this disease. The unique characteristics of *P. destructans* also suggest that collaboration among researchers in the “separate” fields of fungal pathogenesis of plants and animals has the potential to answer new questions raised on how pathogens can emerge from distantly related niches or kingdoms.

## Data Availability

No data was generated in this work.
